# Prediction of Pile Shaft Capacity in Tension Based on Some Direct CPT Methods—Vistula Marshland Test Site

**DOI:** 10.3390/ma15072426

**Published:** 2022-03-25

**Authors:** Łukasz Zwara, Lech Bałachowski

**Affiliations:** Department of Geotechnical and Hydraulic Engineering, Faculty of Civil and Environmental Engineering, Gdańsk University of Technology, G. Narutowicza 11/12, 80-233 Gdańsk, Poland; lech.balachowski@pg.edu.pl

**Keywords:** cone penetration test, tension pile, shaft resistance, sleeve friction, static load test

## Abstract

This paper presents different CPT methodologies for the prediction of the pile shaft resistance in tension on the example of three reference screw piles of the Jazowa test site in Poland. The shaft capacity was estimated based on the cone resistance, sleeve friction and CPT excess pore water pressure. Three piles with a diameter of 0.4 m and the length varied from 8 m to 14.6 m were subjected to static load tests in tension. Their results were used to determine the ultimate bearing capacity of the reference piles. The pile shaft resistance was estimated according to the AFNOR standard, Doan and Lehane 2018 centrifuge tests based method (Delft University of Technology approach), the Modified Unicone method, KTRI (Kajima Technical Research Institute) and LCPC (Laboratoire Central des Ponts et Chaussées) method. Then, the ultimate bearing capacity determined in static load tests was compared to the estimated values according to five different methods. The best estimation, fitting almost perfectly to static load test values, was obtained with the AFNOR method, whereas the other predictions significantly underestimated the ultimate bearing capacity.

## 1. Introduction

The Eurocode 7 recommends performing some in situ tests including cone penetration test, pressuremeter test or dilatometer tests to estimate pile bearing capacity in the direct design approach. It considers direct design methods where the pile bearing capacity is calculated using the results of in situ soil investigation. The main methodologies of pile bearing capacity prediction are based on CPT tests. The modern pile design codes use the similarity in the installation process of displacement piles and cone penetration test where large displacements are induced in the surrounding soil mass and at the contact between pile shaft/friction sleeve and the soil. The earliest and most common methods which appeared in the scientific world are, for example, the Aoki and de Alencart (1975) method [1], Schmertmann method (1978) [2], de Ruiter and Beringen (1979) method [3] or Bustamante and Gianeselli (1982) (LCPC) [4], which were based on cone resistance. The degradation of pile–soil interface parameters with large displacements are observed with so-called friction fatigue. This phenomenon was studied in the case of driven and pushed-in instrumented piles with normal stress and lateral friction evolution registered during pile installation and subsequent loading [5]. The degradation of the pile–soil interface parameters can be also modeled in large displacement ring shear apparatus or even direct shear test [6]. The sleeve friction registered in the cone penetration test takes into account the remolded soil strength, so it includes to some extent the effect of soil disturbance due to pile installation. The design methods using sleeve friction are examined in this study.

Nowadays, the CPT-based methods are widely used for direct and undirect pile design. In the first approach, an analogy between the CPT cone and pile base resistance is quite evident. One should however take into account the size effect, soil layering and the installation effect related to the pile technology. The determination of pile skin friction based on CPT results is not straightforward. It is generally based on some empirical correlations with cone resistance and less frequently on the relationships with sleeve friction and mobilized water pressure. One should also consider the installation effects concerning stress state and soil density which could significantly modify the pile response in case of large displacement piles. This subject was discussed in many papers such as, for example, Eslami and Fellenius (1997) [7] or Doan and Lehane (2018) [8,9,10].

One should also note that current CPT-based methods were elaborated for typical cohesive or cohesionless soils and their application in intermediate soils is questionable due to partial drainage conditions between fully drained and undrained behavior. Moreover, organic soils considered in the present study are not included in the typical pile design methods. Another question is related to the CPT soil classification system–based on soil type behavior–in which some organic soils are presented as intermediate soils. The limited applicability of CPTu-based classifications for organic soils at this site was already argued by Bałachowski et al. (2019) [11]. Thus, the novelty of the present approach considers the check of new design methods in organic soils not properly classified by CPT soil classification systems.

The pile bearing capacity can be determined using static load tests which are the most accurate methods for predicting pile capacities. The interpretation of load-settlement curves from static load tests and determination of ultimate bearing capacity can be performed using different approaches such as the Chin, Brinch Hansen, Davisson or DeBeer methods [12]. It is admitted in the Eurocode that the ultimate bearing capacity of the pile determined from the static load test corresponds to the head displacement equal to 10% of the pile diameter. In this paper, the static load curves were interpreted with the Chin method and extrapolated to 10% of the pile diameter.

The novelty of the present approach is to include in the pile bearing capacity calculation not only cone resistance but the sleeve friction and pore water pressure mobilized during CPT penetration as well. Moreover, the soil behavior type derived from CPT was used to classify the soil and to calculate skin friction according to empirical formulae based on model tests in centrifuge or static pile load tests. In this way, the appropriate formula chosen to calculate skin friction can be directly related to the CPT soil classification system. This new approach was used for the modern technology of screw displacement piles applied in organic soil conditions including organic clay, mud and peat layers [11]. One of these soil classification systems, used in this study, is the Robertson chart [13].

In this paper, the bearing capacity of tension screw piles subjected to uplift loading will be considered. The pile bearing capacity is estimated using five CPT-based methods: the AFNOR method, the Doan and Lehane approach, the Modified Unicone method, KTRI, LCPC and based on the static load test. The bearing capacity of screw piles in compression using the AFNOR approach was already examined at this test site by Bałachowski et al. (2019) [14].

## 2. Materials and Methods

### 2.1. Soil Profile

**Figure 1:**
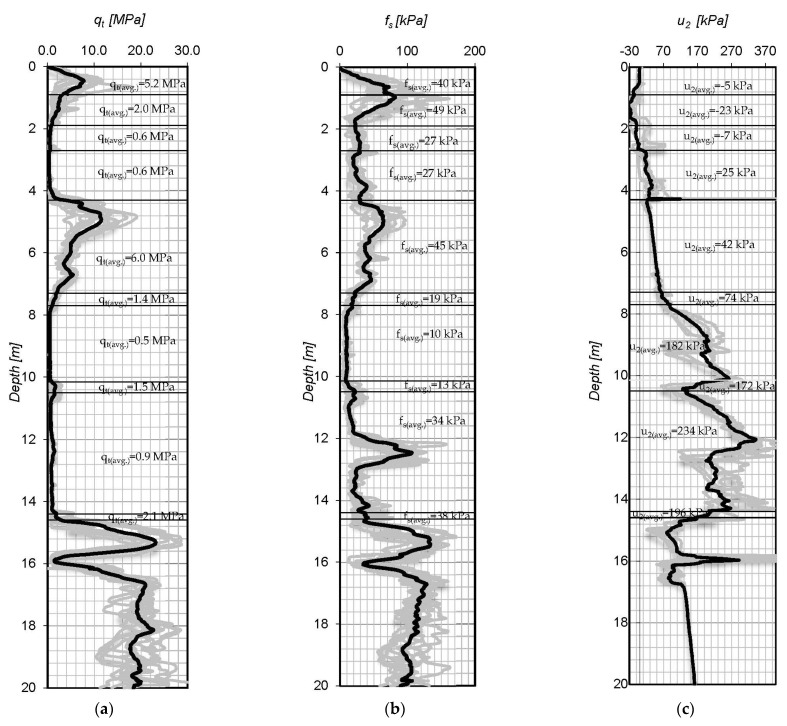
The CPTu measurement results of: (**a**) corrected cone resistance, (**b**) sleeve friction, (**c**) pore water pressure with average CPTu measurement value per soil layer.

The Jazowa test site is situated on the Vistula Marshlands. CPT tests were conducted on two testing fields in proximity of the S-7 highway in order to establish the geotechnical documentation. CPT measurements were carried out down to 20 m. The data were recorded at intervals of 2 cm. Then, the readings were filtrated and the results of 10 CPT soundings were averaged. The measurements were performed using a standard cone. The CPTs results are presented in Figure 1.

### 2.2. Soil Identification

The soil identification was performed based on the normalized cone penetration test results. The layers of the soil profile were determined using the Robertson method [13] by application of the following formulas:

(1) $$I_{c}={\left({\left(3.47-logQ_{tn}\right)}^{2}+{\left(logF_{r}+1.22\right)}^{2}\right)}^{0.5}$$

where *I_c_* is the soil behavior type index, *n* is the stress exponent, *Q_tn_* is the normalized cone resistance and *F_r_* is the normalized friction ratio.

The soil behavior type index, *I_c_*, is used to determine the type of soil according to the Robertson CPT-based classification [13].

The normalized cone resistance was given by equation [13]:

(2) $$Q_{tn}=\left(q_{t}-\sigma_{v0}/P_{a}\right)\times {\left(P_{a}/\sigma_{v0}^{\prime }\right)}^{n}\;$$

where *q_t_* is the corrected cone resistance, *σ_v_*_0_ is the total vertical stress, $\sigma_{v0}^{\prime }$ is the effective overburden stress and *P_a_* is the atmospheric pressure (100 kPa).

The corrected cone tip resistance was calculated as:

(3) $$q_{t}=q_{c}+u_{2}\times \;\left(1-a\right)$$

where *q_c_* is the measured CPT cone tip resistance and *u*_2_ is the measured pore water pressure at cone shoulder and *a* is the net area ratio of the cone equal to 0.8.

The total vertical stress is given by the equation:

(4) $$\sigma_{v0}=\sum \left(z_{i}\times \gamma_{i}\right)$$

where *z_i_* is the penetration depth and *γ_i_* is the unit weight of the soil at the given level.

The effective overburden stress is given by the equation:

(5) $$\sigma_{v0}^{\prime }=\sigma_{v0}-u$$

where *u* is the in situ pore water pressure.

Normalized friction ratio *F_r_* is defined as:

(6) $$F_{r}=\left[f_{s}/\left(q_{t}-\sigma_{v0}\right)\right]\;\times \;100\%$$

The unit weight of the soil is given by equation [15]:

(7) $$\gamma =\left(0.27\;\times \;\left(logR_{f}\right)+0.36\;\times \;\left[log\left(q_{t}/P_{a}\right)\right]+1.236\right)\;\times \;\gamma_{w}$$

where *γ_w_* is the unit weight of water.

Friction ratio *R_f_* is defined as:

(8) $$R_{f}=\left(f_{s}/q_{t}\right)\;\times \;100\%$$

The stress exponent is defined as [13]:

(9) $$n=0.381\times I_{c}+0.05\times \;\left(\sigma_{v0}^{\prime }/P_{a}\right)\;-\;0.15$$

where *n* varies from 0.5 for sand to 1.0 for clay.

The Jazowa soil profile established according to the Robertson chart [13] was determined using *I_c_*.

The Modified Unicone method defines the types of soil based on the Modified UniCone chart [16]. The chart determines 11 soil types by plotting the effective cone resistance *q_E_* against the sleeve friction, *f_s_*, obtained from the CPT test. The effective cone resistance is defined as follows [16]:

(10) $$q_{E}=q_{t}\;-\;u_{2}$$

For each soil type, the method attributes an average pile shaft coefficient, *C_se_*, which is defined as the ratio of pile unit shaft friction and the effective cone resistance. The *C_se_* values are indicated on the Modified Unicone chart [16].

According to the Modified Unicone approach, the pile shaft coefficient is a logarithmic function of the soil behavior index, *I_c_*, calculated using the Formulas (1), (2) and (9).

### 2.3. Calculation Methods

There is a considerable portion of organic soils in the analyzed profile. They are classified as intermediate soils according to considered CPT soil classifications. “There is not a universally accepted method for estimation of the unit shaft friction of displacement piles in intermediate soils such as silts and clayey sands” [8]. The intermediate soils present characteristics which make the pile shaft capacity estimation based on CPT measurements much more complicated than the CPT result interpretation for sand and clay, as the cone penetration performed with a standard rate is usually drained or undrained in these materials, respectively [9]. The behavior of intermediate soil during in situ investigation is directly related to partial drainage which can easily lead to misinterpretation of the pile shaft capacity evaluation. The following methods were selected in order to present different approaches for pile shaft capacity predictions. These approaches are based on correlations between laboratory and in situ tests. The presented methods evaluate the shaft resistance using CPT values and complex parameters in opposite to straightforward conservative methods based on constants depending on cone tip resistances *q_c_* maximum and minimum values. The applicability of the CPTu test in excess water pressure monitoring during displacement piles construction at this site was presented in Bałachowski et al. (2021) [17]. Thus, the conservative methods can heavily underestimate the pile shaft resistance as the soil behavior analysis based on CPT results is strongly simplified. Therefore, the main objective of this paper is to present the basis of some recent pile shaft bearing capacity prediction methods and to discuss the discrepancies on the example of the local geotechnical conditions.

#### 2.3.1. AFNOR Standard Methodology

The AFNOR standard [18] proposes two methods to evaluate the pile shaft resistance. The first approach is based on pressuremeter measurements and the second one is based on penetrometer tests. The French norm distinguishes 8 classes of piles distributed into 20 categories. The calculation models depend on the pile’s class and the pile’s category. For the penetrometer model, the unique measurement value necessary to evaluate the pile shaft bearing capacity is the cone tip resistance.

In this research, the reference piles according to the AFNOR standard, belong to the 3rd class “Screw piles” and to the 7th category “Screw cast-in-place pile”.

The AFNOR standard proposes the following equation with maximum cone tip resistance condition to calculate the unit shaft pile resistance:

(11) $$q_{s}=\alpha \times f_{sol}$$

where *α* is an installation factor and *f_sol_* is a soil-dependent function. The installation factor is a constant which depends on the soil type and pile class. The soil-dependent function is defined as follows:

(12) $$f_{sol}=\;\left(a\times q_{c}+b\right)\times (1-e^{\left(-c\times qc\right)})$$

where *a*, *b* and *c* are parameters based on the AFNOR soil classification and *q_c_* is cone tip resistance.

The unit shaft resistance was determined based on the mean cone tip resistance value calculated for each soil layer and using Equations (11) and (12). The total pile shaft capacity was calculated using the following formula:

(13) $$R_{t}=\sum \left(q_{si}\times A_{si}\right)$$

where *q_si_* is skin friction within a soil layer and *A_si_* is an area of the pile shaft. The total pile shaft resistance was calculated as the sum of pile shaft resistances of all layers.

#### 2.3.2. Doan and Lehane Method

Doan and Lehane [8,9,10,19,20] examined the correlations between the corrected cone resistance and unit shaft friction (*β_c_* ratio) for displacement and non-displacement piles. The research was performed in sand, clay and intermediate soils with different fine contents in order to investigate the influence of clay in sand mass on CPT soil response related to different levels of drainage conditions as the standard CPT penetration is usually drained in sands, undrained in clays and partially drained in intermediate soils.

The skin friction during the tension load test in geotechnical centrifuge and steel pressure chambers was evaluated for each investigated soil. The *β_c_* ratio was defined as the average corrected cone tip resistance divided by the peak shaft friction obtained from the tension load test:

(14) $$\beta_{c}=q_{t}/\tau_{f}$$

The obtained *β_c_* values for different types of soil were in good agreement with the corresponding soil behavior type index, *I_c_* [8].

The *β_c_* methodology is based on the principle that the *β_c_* ratio is constant for the sand-like and the clay-like soils and various in logarithmic scale for the transitional-like. The variation is related to the presence of clay fraction which has a strong impact on soil response. The soil behavior type index was calculated using the Formulas (1), (2) and (11).

As presented in the Doan and Lehane investigation, the relationship between the *β_c_* ratio and the soil behavior type index, *I_c_*, was independent of the penetration drainage conditions. The correlations were established for undrained, partially drained and fully drained conditions which allowed us to estimate the pile shaft capacity without analyzing the influence of the partial drainage on the CPT results [9].

For the clay-like soils, the *β_c_* ratio was two times higher for rough piles than for smooth ones. For the sand-like soils, the ratio was about 60% higher for rough piles than for smooth ones, and for the transitional ones, it varied between the sand-like and clay-like soils ratios.

For rough displacement piles, the *β_c_* methodology established the following correlations [8]:

(15) $$\beta_{c}=200\;for\;I_{c}\leq 1.8$$

(16) $$\beta_{c}=10^{\left(3.45–0.65\times I_{c}\right)}\;for\;1.8<I_{c}<3.6$$

(17) $$\beta_{c}=30\;for\;I_{c}\geq 3.6$$

For sand and clay, *β_c_* is a constant and for intermediate soils, *β_c_* is a function of *I_c_* value.

The reference piles used in this study were considered as fully displacement piles with a rough interface. The unit pile shaft resistance, *τ_f_*, was determined for each layer in the soil profile using the formula:

(18) $$\tau_{f}=q_{t}/\beta_{c}$$

where *q_t_* is the average corrected cone resistance of the soil layer and *β_c_* is determined based on the average *I_c_* values which were previously calculated for each layer of the soil profile.

#### 2.3.3. Modified Unicone Method

The Unicone method was established by Eslami and Fellenius (1997). It defines the correlations between 5 types of soil according to the Unicone classification chart based on the CPT measurements and the pile shaft coefficients assigned to the soil numbers. The approach was improved by Niazi and Mayne [16] which developed in the Unicone chart 6 additional soil types in order to refine the pile bearing capacity predictions in the intermediate soils as the abrupt variations in the proposed shaft coefficient values between two adjacent soil zones did not allow for some gradual transitions for intermediate soil types.

The Modified Unicone method distinguishes 11 types of soil. They can be determined using either the Modified Unicone chart or the logarithmic expression linking the shaft coefficient value with the soil behavior type value, *I_c_* (see Formulae (20)). In the present paper, the soil profile and the shaft coefficients for each soil layer were defined using the logarithmic expression.

The pile shaft prediction of the Modified Unicone approach is based on the following correlations:

The pile side coefficient for tension, *C_se_*_(*T*)_, is calculated as:

(19) $$C_{se\left(T\right)}=\theta_{tc}\times \theta_{rate}\times \theta_{pile-type}\times C_{se\left(mean\right)}$$

where *C_se_*_(*mean*)_ is a logarithmic formulae determined from empirical investigations. It is defined as:

(20) $$C_{se\left(mean\right)}=10^{\left(0.732\times I_{c}-3.605\right)}$$

where *I_c_* is the soil behavior type index according to the Robertson chart [13].

The *θ_tc_*, *θ_rate_* and *θ_pile_*_-*type*_ are the adjustments factors depending on loading direction, loading procedure and pile type installation method, respectively.

For loading direction, the adjustment factor, *θ_tc_*, is 0.85 for tension and 1.11 for compression.

For the loading procedure in fine-grained soils where *I_c_* > 2.6, the adjustments factor is 0.97 as the predicted pile shaft resistance of the reference piles is compared with the ultimate one evaluated in MLT (Maintain Load Test). The Modified Unicone method established the MLT adjustment factor by comparison of CPTu results with the ultimate pile shaft bearing capacity.

For pile type installation method, the adjustment factor is 0.84 for bored piles, 1.02 for jacked piles and 1.13 for driven piles.

For the present case of study, the selected adjustment factors are:

*θ_tc_* = 0.85 for tension piles,

*θ_rate_*_(MLT)_ = 0.97 for *I_c_* > 2.6 and *θ_rate_* = 1 for *I_c_* < 2.6,

*θ_pile_*_-*type*_ = 1.13 as fully displacement piles were considered.

Unit skin friction, *q_si_*, is calculated as:

(21) $$q_{si}=C_{se}\times q_{E}$$

where *q_E_* is the effective cone tip resistance (*q_E_* = *q_t_* − *u*_2_).

For the purpose of the pile shaft capacity estimation, *I_c_* and *q_E_* were evaluated for each CPT measurement and then the values were averaged within the limits of the soil layers in order to find the mean *I_c_* and *q_E_* value per layer.

The average unit shaft resistance of the layer was calculated by multiplication of the mean cone tip resistance by the adjustment factors. The tension pile factor and pile-type factor were the same for all layers. The loading procedure factor of the layer depended on the mean *I_c_* value of the layer. The total pile shaft resistance, *R_t_*, was calculated as demonstrated for AFNOR methodology by application of the Formula (13).

#### 2.3.4. KTRI Method

The approach is based on a correlation between the ratio of the unit shaft resistance, *f_p_*, to the sleeve friction, *f_s_*, obtained from CPT test and the excess pore water pressure Δ*u*_2_. The excess pore water pressure is calculated as:

(22) $$\Delta u_{2}=u_{2}\;-\;u_{0}$$

where *u*_2_ is the measured pore water pressure at the cone shoulder and *u*_0_ is the in situ pore water pressure. The correlation was established on bored and driven piles embedded in clay, sand and mixed soils located on different sites in Japan. The unit shaft friction is estimated by application of one of the following relationships [21]:

(23) $$f_{p}=f_{s}\times \;\left(\Delta u_{2}/1250+0.76\right)\;for\;\Delta u_{2}<300\;\mathrm{kPa}$$

(24) $$f_{p}=f_{s}\times \;\left(\Delta u_{2}/200-0.5\right)\;for\;\Delta u_{2}>300\;\mathrm{kPa}$$

The graphical representation of these correlations is shown on the KTRI chart [21].

The analyzed CPT excess pore water pressures of the Jazowa soil profile were not greater than 300 kPa. Thus, the first correlation (23) was used to estimate the shaft capacity of the reference piles.

## 3. Results and Discussion

### 3.1. Static Load Test

**Figure 2:**
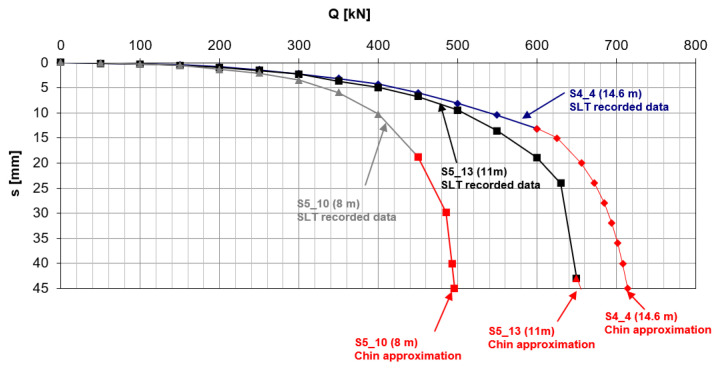
Load–settlement curve of the reference piles.

**Figure 3:**
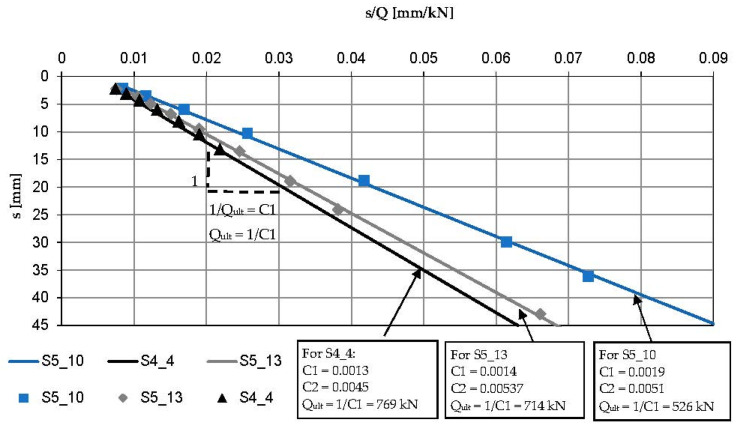
Ultimate bearing capacity in the tension of the reference piles according to Chin’s method.

Within the S-7 project, 69 screw piles were constructed at the test site. There were compression piles, tension piles and anchoring piles used for static load tests. For the purpose of this research, three tension piles were selected: 8 m, 11 m and 14.6 m long (S5_10, S5_13, S4_4). They were chosen as representative ones as they were subjected to maximum head displacement during SLT. The first two piles were floating ones, the longest pile was just resting on the roof of the compacted sand layer. The reference piles were 0.4 m in diameter. They were situated very close to each other within the perimeter of 20 m. They were proof-tested in the tension static load test. The load was applied on the pile head in increments of 50 kN in 10 min intervals and the corresponding pile settlement was recorded. The maximum applied loads were 450 kN for S5_10, 650 kN for S5_13 and 600 kN for S4_4. The pile head displacements under the maximum applied loads were 19 mm (4.8%D), 43 mm (10.7%D) and 13 mm (3.2%D), respectively. The ultimate bearing capacities of the reference piles were estimated by Chin’s approximation as the inverse slope of the linear function f(s) = C_1_.s + C_2_ where s is the settlement of the pile caused by the load, Q, in static load test and C_1_, C_2_ are the Chin’s parameters used to approximate the load–settlement curve. The approximated load–settlement curves of the reference piles with the corresponding SLT results are presented in Figure 2. The ultimate bearing capacity in the tension of the reference piles according to Chin’s method is presented in Figure 3. The maximum applied pile loads varied from 78% of Q_ult_ for S4_4 to 91% of Q_ult_ for S5_13.

### 3.2. Soil Classification

**Figure 4:**
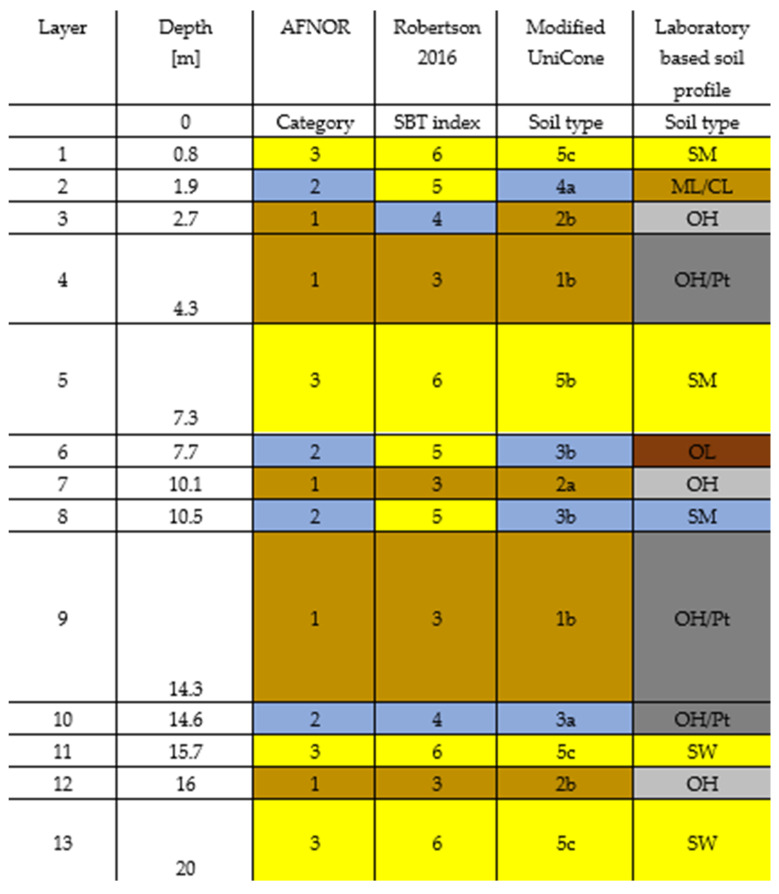
The Jazowa site soil profile according to the presented methodologies. Soil type description of Figure 4: AFNOR category: 1—clayey soil, 2—Intermediate soil, 3—sandy soil. Robertson 2016 SBT index: 3—clay to silty clay, 4—silt mixtures: clayey silt and silty clay, 5—sand mixtures: silty sand, sandy silt, 6—sands: clean sands to silty sands. Modified UniCone soil type: 1b—soft clay and silt, 2a—silty marine and varved clays, 2b—stiff weathered clay, clay till, 3a—firm to medium soft silty clay, 4a—sandy silt, medium dense silt, 5b—Medium dense sand, 5c—dense sand. Laboratory-based soil profile [11]: CL—clay of low plasticity, ML—silt of low plasticity, OH—organic soil of high plasticity, OL—organic soil of low plasticity, Pt—peat, SM—silty sand, SW—well-grade sand.

The Jazowa site soil profile according to the examined methodologies is presented in Figure 4. The three analyzed soil classifications–based on the Robertson SBT index, used in the AFNOR standard and the Modified Unicone method give a similar interpretation of subsoil layers. The sandy soils according to the AFNOR standard were defined as sand by the Robertson classification and medium dense sand and dense sand by the Modified Unicone method. The clayey soil layer locations were consistent for all examined methodologies. Regarding the intermediate soils, the AFNOR standard does not differentiate them in contrast to the Robertson classification and the Modified Unicone method. The intermediate soils of the Jazowa site are defined as sand mixtures and silt mixtures according to the Robertson approach and as sandy silt, medium dense silt, clayey silt and firm to medium soft silty clay according to the Modified Unicone method.

### 3.3. Calculated Pile Shaft Capacity

The shaft capacities of the three reference piles were estimated according to the AFNOR standard, the *β_c_* approach, the Modified Unicone method, KTRI and LCPC. The measured and predicted bearing capacities of the reference piles are presented in Table 1. For all three examined piles the highest pile shaft capacities were predicted by the AFNOR methodology. One should however note that the calculated values of shaft resistance slightly overpredict the results of static load tests. The second strongest values were estimated using the LCPC approach, but in this case, all the calculated values are on the safe side, i.e., they are smaller than the SLT results. One should also note that the results obtained with the *β_c_* approach are only slightly smaller than calculated according to the LCPC method. For instance, for the 14.6 m pile (S4_4), the shaft resistance determined with the *β_c_* approach was only 2% smaller than estimated according to the LCPC method. For the 8 m pile (S5_10) and 11 m pile (S5_13), the pile shaft resistance determined with the *β_c_* approach and the Modified Unicone method were roughly the same. For all reference piles, the smallest values of pile capacity were estimated using the KTRI method.

### 3.4. Comparison between the Predicted and Ultimate Shaft Resistance

The predicted bearing capacities for tension piles were compared to the ultimate ones from the static load tests. As indicated in Table 2, for the 11 m pile (S5_13) and 14.6 m pile (S4_4), the predicted values based on the AFNOR methodology fitted almost perfectly to the measured loads. For both piles, the AFNOR methodology showed the predicted bearing capacity in a range of 6% of Q_ult_ from the proof-test. For S5_10, the AFNOR methodology overestimated the shaft resistance by 12%. For S4_4, the estimations of the *β_c_* approach, the Modified Unicone method and LCPC were quite accurate reaching from 76% to 82% of the measured bearing capacity. For S5_10 and S5_13, the LCPC results underestimated the pile shaft capacity by 22% and 32%, respectively. Regarding the other estimations for these piles, they represented around 60% of Q_ult_ according to the *β_c_* approach and the Modified Unicone method and around 50% of Q_ult_ according to the KTRI.

### 3.5. Discussion

The uplift bearing capacity of three piles constructed in practically the same soil conditions was examined. The shaft bearing capacity based on the AFNOR standard was predicted almost perfectly for two reference piles. For the third (the shortest one), the predicted value was 12% higher than the ultimate one obtained from the static load test, which was still a quite good estimation compared to the other studied methodologies. For the 14.6 m pile (S4_4), the AFNOR method, the Modified Unicone method and the LCPC underestimated the pile shaft capacity by around 20%. For S5_10, the LCPC underestimated the shaft resistance in the same range as for S4_4. For the *β_c_* approach, the Modified Unicone method and the KTRI, a significant underestimation of the pile shaft bearing capacity was observed for floating piles S5_10 and S5_13. These predicted values were from 55% to 40% lower than the measured ones. One should note that the methods based on cone resistance provide a better estimation of pile bearing capacity than those taking into consideration sleeve friction value directly as KTRI or in an indirect way, such as the Modified Unicone method. This observation considers screw displacement piles in soft organic soils encountered in this study.

## 4. Conclusions

This study estimated and analyzed the pile shaft bearing capacity of the three reference piles from the Jazowa site. They were embedded in practically the same soil conditions including soft organic soils and sandy layers. These subsoil conditions are commonly present on the Vistula Marshland site. The pile shaft resistance was evaluated based on CPTs measurements according to five different methodologies. The soil layering was determined on CPT soil classification charts. One should note a good agreement between the soil types determined with different classifications. The predicted shaft friction was compared with the ultimate pile shaft capacity obtained from the static load test. Based on the present investigation, the following conclusions can be drawn for the considered soil conditions:

- Common methods of shaft resistance prediction underestimate the pile capacity in tension;
- The AFNOR standard evaluates accurately the shaft resistance in tension. Two AFNOR predictions were almost perfect and the third one slightly overestimated the pile resistance;
- AFNOR determines the highest shaft resistance factors for sand and intermediate soils amongst the analyzed methodologies. The shaft resistance in sand layers according to AFNOR was significantly higher than that evaluated by other methodologies;
- Unicone defines the highest shaft resistance factors for clayey soils. However, these factors are just slightly higher than the ones determined by other methodologies. As a consequence, the shaft resistance values in clayey soils are comparable for each methodology;
- Shaft resistance evaluation based on pore water pressure mobilization and sleeve friction values measured by penetrometer does not correctly predict the pile capacity for relatively low mobilized pore water pressure values not exceeding 150 kPa.

The present research confirms a very good performance of the AFNOR method for the geotechnical conditions of the Vistula Marshland. Nevertheless, as only three reference piles were analyzed, an investigation of a greater number of reference piles is necessary to confirm that the AFNOR method is ideal for the geological conditions of the Vistula Marshland.

In this study, the organic soils were classified as intermediate ones in the considered soil classification systems. Further studies are necessary to include different types of organic soils in the soil classification systems and to relate them with pile design methods.

## Figures and Tables

**Table 1:** Measured and predicted bearing capacities of the reference piles.

Pile Number	Q_ult_ [kN]	Q_SLT_/Q_ult_ *R_t_* [kN]	AFNOR *R_t_* [kN]	*β_c_* Approach *R_t_* [kN]	Modified Unicone *R_t_* [kN]	KTRI *R_t_* [kN]	LCPC *R_t_* [kN]
S5_10	526	86%	590	318	320	282	408
S5_13	714	91%	673	421	405	332	485
S4_4	769	78%	812	617	583	420	631

**Table 2:** Ratio between predicted and ultimate pile shaft capacity, *R_t_*.

	Reference Pile		
	S5_10 (8 m Pile)	S5_13 (11 m Pile)	S4_4 (14.6 m Pile)
Methods	*R_t_* [kN]	*R_t_* [kN]	*R_t_* [kN]
AFNOR	1.12	0.94	1.06
*β_c_* approach	0.61	0.59	0.80
Modified Unicone	0.61	0.57	0.76
KTRI	0.54	0.45	0.60
LCPC	0.78	0.68	0.82

## Data Availability

Data are contained within the article.
